# Neurophysiological Markers of Emotion Processing in Burnout Syndrome

**DOI:** 10.3389/fpsyg.2017.02155

**Published:** 2017-12-13

**Authors:** Krystyna Golonka, Justyna Mojsa-Kaja, Katarzyna Popiel, Tadeusz Marek, Magda Gawlowska

**Affiliations:** ^1^Institute of Applied Psychology, Jagiellonian University, Krakow, Poland; ^2^Department of Cognitive Neuroscience and Neuroergonomics, Institute of Applied Psychology, Jagiellonian University, Krakow, Poland; ^3^Neurobiology Department, The Malopolska Centre of Biotechnology, Jagiellonian University, Krakow, Poland

**Keywords:** burnout, neurophysiological markers, emotion processing, ERPs, N170, VPP, EPN, LPP

## Abstract

The substantial body of research employing subjective measures indicates that burnout syndrome is associated with cognitive and emotional dysfunctions. The growing amount of neurophysiological and neuroimaging research helps in broadening existing knowledge of the neural mechanisms underlying core burnout components (emotional exhaustion and depersonalization/cynicism) that are inextricably associated with emotional processing. In the presented EEG study, a group of 93 participants (55 women; mean age = 35.8) were selected for the burnout group or the demographically matched control group on the basis of the results of the Maslach Burnout Inventory – General Survey (MBI-GS) and the Areas of Worklife Survey (AWS). Subjects then participated in an EEG experiment using two experimental procedures: a facial recognition task and viewing of passive pictures. The study focuses on analyzing event-related potentials (ERPs): N170, VPP, EPN, and LPP, as indicators of emotional information processing. Our results show that burnout subjects, as compared to the control group, demonstrate significantly weaker response to affect-evoking stimuli, indexed by a decline in VPP amplitude to emotional faces and decreased EPN amplitude in processing emotional scenes. The analysis of N170 and LPP showed no significant between-group difference. The correlation analyses revealed that VPP and EPN, which are ERP components related to emotional processing, are associated with two core burnout symptoms: emotional exhaustion and cynicism. To our knowledge, we are one of the first research groups to use ERPs to demonstrate such a relationship between neurophysiological activity and burnout syndrome in the context of emotional processing. Thus, in conclusion we emphasized that the decreased amplitude of VPP and EPN components in the burnout group may be a neurophysiological manifestation of emotional blunting and may be considered as neurophysiological markers of emotional exhaustion and cynicism. Additionally, we did not observe a decrease in LPP, which may be considered as a marker that significantly differentiates burnout from depression.

## Introduction

Studies on burnout syndrome depict the typical characteristics and consequences of long-term work-related stress. Burnout has an impact on the mental and physical health of employees (for a review, see Schaufeli and Enzmann, 1998); as a consequence, it has the serious socio-economic impact of decreased productivity levels, increased rates of resignation and premature retirement (Schaufeli et al., 2009). Therefore, there is a need for empirical research that provides answers related to the neuronal mechanisms of burnout and further implication of this knowledge in interventive programs. Burnout has three main components: a state of emotional exhaustion, depersonalization or cynicism and lower personal accomplishment (Maslach and Leiter, 1997, 2008). The existing studies on burnout analyze the negative effects of long-term work-related stress on individuals’ functioning in terms of the complex emotional and cognitive consequences (e.g., Maslach and Leiter, 1997, 2008; Schaufeli and Greenglass, 2001; Sandström et al., 2005; Van der Linden et al., 2005; Schmidt et al., 2007; Oosterholt et al., 2012, 2014; Deligkaris et al., 2014; Golkar et al., 2014). Together, the cognitive and emotional dysfunctions caused by long-term stress can lead to impaired ability to regulate emotional tension and cope with stressors. Studying the relationship between emotional exhaustion and emotional regulation in burnout subjects, Golkar et al. (2014) observed that burnout individuals have impaired ability to downregulate negative emotions, which can lead to an increased susceptibility to depression.

Besides the commonly used subjective measures, in burnout research there is a limited number of studies dedicated to behavioral (e.g., Sandström et al., 2005; Oosterholt et al., 2012; Bianchi and Laurent, 2015), neurophysiological (Luijtelaar et al., 2010; Golkar et al., 2014; Sokka et al., 2014, 2017; Golonka et al., 2017) and neuroimaging outcomes (Durning et al., 2013; Golkar et al., 2014; Tei et al., 2014).

Neurophysiological and neuroimaging studies provide evidence for impairments in cognitive and emotional processing in burnout that helps broaden our knowledge of the mechanisms underlying emotional exhaustion and depersonalization/cynicism, which are the core components of burnout. Studies that employ functional magnetic resonance imaging (fMRI) have indicated that burnout is associated with changes in functional brain anatomy. For example, Durning et al. (2013) observed that higher burnout scores are associated with reduced functional activation in the right dorsolateral prefrontal cortex (rDLPFC), the medial frontal gyrus (MFG) and increased activation of the right posterior cingulate cortex (rPCC) during performance of cognitive tasks. Tei et al. (2014) revealed that burnout severity is explained by decreased empathy-related brain activity, proving that emotional functioning is altered in burnout groups. Golkar et al. (2014) observed that functional connectivity between the amygdala and the anterior cingulate cortex (ACC) is weaker in burned-out subjects; this is in contrast to stronger connectivity from the amygdala to the cerebellum and the insular cortex, which may have further consequences for regulating mechanisms and reactive and proactive control in burnout subjects (Golonka et al., 2017).

In addition to fMRI, other neuroimaging techniques, such as analyzing event-related potentials (ERPs) in EEG recordings, enable investigation of the time course of this information processing in the brain. Existing studies on ERP components in burnout are still very limited (i.e., Luijtelaar et al., 2010; Sokka et al., 2014, 2017; Golonka et al., 2017), but their findings are promising and contribute to the description of the neural mechanisms underlying burnout syndrome.

### Emotional Consequence of Burnout

Burnout is inextricably linked to significant emotional consequences. Core burnout components are *emotional exhaustion* and specific negative emotions and attitudes toward work (labeled as *cynicism*) and other people (labeled as *depersonalization*). These aspects are well recognized in research that employed subjective measures (i.e., Maslach et al., 1996; Maslach and Leiter, 1997, 2004, 2008; Mojsa-Kaja et al., 2015).

Burnout syndrome is associated with emotional exhaustion and the tendency to experience negative emotions. Additionally, emotional disengagement and distancing oneself from work and co-workers/clients/patients, etc. may be seen as an adaptive defense mechanism that helps in coping with difficult work-related circumstances. Hypothetically, this defense mechanism may reduce the anxiety associated with, for example, an overwhelming workload, a lack of control, insufficient reward, an unsupportive community, unfairness, conflicting values, etc.; however, it usually leads to further problems due to subsequent social and job consequences (e.g., deterioration in interpersonal relations and lower professional efficacy). Even if the attitude typical of depersonalization/cynicism brings temporal profits, it lacks long-lasting benefits.

Taking into account the state of emotional exhaustion, depletion and decline in individual functioning in burnout, we hypothesized that emotion processing in burnout subjects would differ, compared to a control group.

### Emotion Processing and Its Neurophysiological Indicators

Regarding the strong emotional component in burnout syndrome, the processing of emotion-related information remains particularly interesting. Due to the high adaptive importance of emotional stimuli, they seem to interfere with other kinds of stimuli (Ramos-Loyo et al., 2013). For the efficient functioning (defined as fast and competent reactions) which is crucial in a social and work environment, it is particularly important to direct attentional resources to the most important stimuli. A very complex and demanding work environment usually continuously exploits cognitive and emotional resources. Regarding long-term work-related stress, the effect of overstimulation and work overload may significantly impair the functioning of the individual.

In the presented study, we focus on analyzing the processing of affect-related information based on the well-documented ERP indicators of emotional information processing (e.g., Eimer and Holmes, 2002; Batty and Taylor, 2003; Schupp et al., 2003; Moser et al., 2006; Olofsson et al., 2008), compared between two groups: burnout subjects and healthy controls.

Research on emotion processing typically implements two types of stimuli: images of emotional facial expressions and natural scenes (Wangelin et al., 2012). In our study, we implement both these types of stimuli to test whether burnout symptoms are related to altered or impaired response to affect-related events. We assumed that the state of emotional depletion might be related to blunted neurophysiological response to a specific type of information—stimuli that have emotional valence. Thus, our main hypothesis states:

H: Burnout subjects demonstrate a weaker response to affect-evoking stimuli.

### ERPs Related to a Facial Recognition Task: N170 and VPP

The mechanism of face perception has been broadly studied in brain research. Facial expression is a fundamental stimulus that conveys socially and emotionally relevant information that is critical for adaptive functioning in social environments. Given this crucial significance of faces, it is not surprising that the study of human face processing is one of the most intensively explored areas in visual cognition and emotion research.

The ERP components associated with face perception have been described in many experiments. A substantial body of research shows that pictures of faces evoke a larger ERP of negative polarity between 130 and 200 ms than other objects (Rossion and Jacques, 2008; Eimer, 2011). This ERP component peaks at occipitotemporal electrode sites about 170 ms after stimulus onset and has been called the N170 (Bentin et al., 1996). According to Schweinberger (1996), N170 is reduced if the face is perceptually degraded. N170 is temporally congruent with a positive-going ERP component and has maximum amplitude over central scalp electrodes; this is called vertex positive potential (VPP) (Bötzel and Grüsser, 1989). Joyce and Rossion (2005) stated that N170 and VPP have the same brain generators and observed that their amplitudes fluctuate conversely. These two ERP components show similar properties, namely greater sensitivity to faces than to other types of stimuli (Joyce and Rossion, 2005; Eimer, 2011).

Abnormalities in face processing measured by VPP and/or N170 have been studied in a wide range of psychiatric and neurological disorders, including bipolar disorder, depression, social phobia, schizophrenia, autism spectrum disorders and Parkinson’s disease (for a review, see Feuerriegel et al., 2015). Despite some inconsistencies in findings, the growing body of research shows that these ERPs might play the role of neurophysiological markers that reflect social impairments in these disorders. For example, Foti et al. (2010) showed that in major depressive disorder, electrocortical response to emotional faces indexed by VPP was absent.

No studies have yet investigated VPP/N170 modulation by burnout. Therefore, our hypotheses are based on the close relation between burnout and depressive symptoms. As the study sample is not clinical, we expect to observe a significant decline in VPP amplitude:

H1: Burnout subjects present declined VPP amplitude to emotional faces.

As it is assumed that N170 and VPP are not only evoked by the same brain source, but also have converse fluctuation and demonstrate identical functional properties (Joyce and Rossion, 2005), no additional hypotheses on N170 were formulated.

### ERPs Related to Passive Picture Viewing: EPN and LPP

Many studies have examined ERP responses to complex images from the International Affective Picture System (IAPS). IAPS consists of stimuli standardized for the basic dimensions of emotion (arousal and valence) which categorize stimuli as pleasant, neutral, or unpleasant; these are used to study both emotion and emotion regulation in adults (Lang et al., 1999; Olofsson et al., 2008).

When analyzing processing of affective pictures, there are two main ERP components which are sensitive to motivational salience of stimuli: early posterior negativity (EPN) and late positive potential (LPP). Moreover, their amplitude variance has been linked with the changes in allocating attentional resources (Schupp et al., 2000, 2006, 2012; Hajcak and Dennis, 2009). It has been consistently observed that EPN and LPP amplitudes increase when stimuli are perceived as more significant; this was observed in processing pleasant and unpleasant compared to neutral IAPS stimuli (Schupp et al., 2006; Hajcak and Dennis, 2009).

The EPN component is observed between 150 and 350 ms; the LPP component is observed between 300 and 700 ms over centroparietal regions (Schupp et al., 2000, 2006). Sabatinelli et al. (2013) analyzed the associations between early and late ERPs using functional MRI signals of cortical and subcortical brain areas. They found that emotional modulation of the LPP correlates with subcortical and visual cortical activation; emotional modulation of the EPN only (and modestly) correlates with subcortical and corticolimbic brain areas. These findings suggest that EPN may refer to motivational relevance, while LPP refers to emotional discrimination. Schupp et al. (2004) point out that, at the functional level, enhanced EPN reflects enhanced perceptual processing and may be mediated by the amygdala activity generated by emotional pictures. Regarding burnout subjects’ disengagement, emotional blunting and being “uninvolved,” it may be hypothesized that:

H2: Burnout subjects reveal decreased EPN amplitude when processing emotional scenes.

This hypothesis is derived from our general assumption about weaker emotional response in burnout subjects. However, when analyzing the relation between burnout and depression and anxiety (Schaufeli and Greenglass, 2001; Bianchi and Laurent, 2015; van Dam, 2016), a contradictory hypothesis could be formulated. In neuroimaging studies on depression, the decreased regulatory effect of the cortical areas over the limbic regions is emphasized. Positron emission tomography (PET) and fMRI studies have shown decreased activation of cortical regions (Ketter et al., 1996; Drevets et al., 1997; Mayberg et al., 1999) and increased activation of limbic structures (Mayberg et al., 1999; Drevets, 2000; Sheline et al., 2001). Anand et al. (2005) found that depressed patients had greater activation of ACC and limbic regions (amygdala, paleostriatum, and medial thalamus). Additionally, they observed decreased cortico-limbic correlations in depressed subjects, compared to healthy individuals. This may lead to the conclusion that greater amygdala activity elicits greater neurophysiological response and greater EPN. Similarly, anxiety-prone subjects reveal increased activation in the amygdala and insula (Stein et al., 2007), which may also be reflected in higher EPN amplitude. On the other hand, depression may be also related to blunted amygdala response. Thomas et al. (2001) observed that anxiety disorder was associated with augmented amygdala response to fearful faces, compared to the blunted amygdala response in depression.

Moreover, clinical studies have revealed that highly anxious individuals demonstrate greater LPP amplitude during the presentation of negative pictures, while individuals with clinical depression showed a decrease of LPP amplitude (Hajcak, 2012). Regarding burnout’s strong associations with depressive symptoms (emotional exhaustion and cynicism/depersonalization) and taking into account the characteristics of LPP (i.e., sensitivity to negative valence of pictures) we hypothesized that:

H3: Burnout subjects reveal decreased LPP amplitude when processing emotionally negative scenes.

## Materials and Methods

### Participants

The study was conducted on an initial group of 100 participants aged 25–55 years. The inclusion criteria for the study were employee status (currently employed, active day-shift workers with higher education), right-handedness, correct or corrected-to-normal vision, not pregnant, addiction free and no history of neurological or psychiatric diseases. The initial group of 100 participants was selected based on the results of the Maslach Burnout Inventory – General Survey (MBI-GS; Maslach et al., 1996) and the Areas of Worklife Survey (AWS; Leiter and Maslach, 2004). The controls were matched with subjects with high scores on burnout, taking into account the latter’s demographic characteristics.

The non-clinical burnout group had high scores in the exhaustion and cynicism subscales and low scores in the self-efficacy subscale (see **Table 1**) and in at least three of the six AWS subscales: workload, control, reward, community, fairness, and values. Low scores in AWS reflected a larger mismatch between individuals’ values, needs and work environment. This scale was introduced as a controlling tool to ensure that deterioration in subjective well-being was linked to work-related stress. Inversely, the second group had lower scores in the exhaustion and cynicism subscales, higher scores in the self-efficacy subscale, and medium to high scores in AWS.

**Table 1:** The means (M) and standard deviations (SD) for the burnout and control groups on burnout symptoms (exhaustion, cynicism, efficacy), work-life areas, age and independent-sample *t*-test between burnout and controls.

	Burnout (*N* = 47)	Control (*N* = 46)	*t-value (df = 91)*
	*M (SD)*	*M (SD)*	
**AGE**			
	37.28 (7.75)	34.78 (8.57)	1.47
**MBI-GS**			
Exhaustion	4.17 (0.97)	1.93 (0.72)	12.62^∗∗∗^
Cynicism	4.04 (0.83)	1.47 (0.57)	17.49^∗∗∗^
Efficacy	3.32 (1.08)	4.62 (0.62)	-7.11^∗∗∗^
**AWS**			
Workload	2.28 (0.80)	3.21 (0.82)	-5.49^∗∗∗^
Control	2.57 (0.98)	3.45 (0.67)	-5.00^∗∗∗^
Rewards	2.48 (0.74)	3.43 (0.61)	-6.77^∗∗∗^
Community	2.72 (0.95)	3.63 (0.66)	-5.40^∗∗∗^
Fairness	2.00 (0.66)	3.09 (0.55)	-8.69^∗∗∗^
Values	2.76 (0.66)	3.64 (0.55)	-6.99^∗∗∗^


*Asterisks denote significant difference (^∗∗∗^*p* < 0.001).*

Eventually, due to the poor quality of the EEG data (i.e., extensive muscle and ocular artifacts), 93 subjects were included in the study sample. The results of the burnout group (*N* = 47; 28 women) and the demographically matched control group (*N* = 46; 27 women) were analyzed. The descriptive statistics of the burnout and control groups are presented in **Table 1**, including burnout symptoms, work-life areas and independent *t*-tests with *p*-values. The burnout group varied significantly from the control group, scoring higher on exhaustion and cynicism and significantly lower on efficacy and all AWS dimensions.

This study was carried out in accordance with the recommendations of the APA Ethics Code. All subjects gave written informed consent in accordance with the Declaration of Helsinki. The study protocol was approved by the Bioethics Commission at Jagiellonian University. The subjects were paid for participation in the study.

### Experimental Procedure

Participants were presented with two experimental tasks, prepared and generated using E-Prime 2.0 ©(Psychology Software Tools): a facial recognition task and viewing of passive pictures. Stimuli were presented on a 17″ LCD monitor placed at a viewing distance of approximately 60 cm.

**FIGURE 1:**
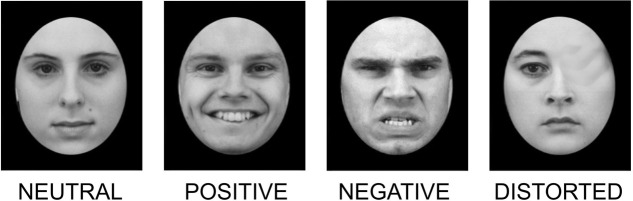
Sample stimuli used in the face recognition task.

In the first task, based on the procedure by Schacht and Sommer (2009), each trial consisted of an oval-shaped black and white face presented on a black background. All the faces were balanced according to their color and tone properties. Stimuli were prepared based on faces from the NimStim dataset (Tottenham et al., 2009). The presented faces fall into one of four categories depending on their visual features: faces with either neutral, negative, or positive emotional expression and faces with a morphological distortion (e.g., a missing eye or nose) (**Figure 1**). To ensure participants’ engagement in the task, they were presented with a masking instruction to assess whether the face was morphologically correct (“press 1”) or distorted (“press 2”). The task consisted of four blocks of 64 stimuli each. All stimuli were presented until the response was submitted; then, a blank screen with a fixation cross was displayed for 1,000–2,000 ms, mean 1,500 ms (Schacht and Sommer, 2009).

For the second task, 120 pictures were selected from the International Affective Picture System (IAPS; Lang et al., 1999): 40 depicted pleasant scenes (e.g., kittens and picturesque scenes), 40 depicted neutral scenes (e.g., cup and chair), and 40 depicted unpleasant scenes (e.g., mutilated bodies and dead animals)^1^. Prior to each picture, a white fixation cross was presented on a black screen for 1,000 ms. Each picture was then displayed in color for 2,000 ms.

After completion of experimental tasks, participants were asked to rate the pictures they had been presented with (both IAPS and faces) on a scale from 1 to 9, where 1 corresponded to very negative, 5 to neutral, and 9 to very positive.

### Data Recording and Acquisition

EEG data was recorded and processed in accordance with guidelines for EEG studies (Keil et al., 2014). Continuous dense-array EEG data (HydroCel Geodesic Sensor Net, EGI System 300; Electrical Geodesic Inc., Eugene, OR, United States) was collected from a 256 channel EEG at a sampling rate of 250 Hz (band-pass filtered at 0.01–100 Hz with a vertex electrode as a reference) and recorded with NetStation Software (Version 4.5.1, Electrical Geodesic Inc., Eugene, OR, United States). The impedance for all electrodes was kept below 50 kΩ. The offline data analysis was conducted with the open source EEGLAB toolbox^2^ (Delorme and Makeig, 2004). Data was digitally filtered to remove frequencies below 0.5 Hz and above 35 Hz. Average reference was recomputed and bad channels were automatically removed by kurtosis measures with a threshold value of 5 standard deviations. Next, continuous data was visually inspected to remove remaining artifactual data manually, i.e., channels or time epochs containing high-amplitudes, high-frequency muscle noise, and other irregularities.

Independent component analysis was used to remove artifacts from data. Due to the large number of channels, decomposition of EEG data with the Infomax algorithm was preceded with Principle Component Analysis. Fifty independent components were extracted and visually inspected for each subject. Based on the spatiotemporal pattern (Bell and Sejnowski, 1995; Jung et al., 2000), components recognized as blinks, heart rate, saccades, muscle artifacts, or bad channels were removed. Missing channels were interpolated and ICA weights recomputed.

For both tasks, the EEG was segmented for each trial beginning 200 ms before each stimulus onset and continuing for 1,200 ms (i.e., for 1,000 ms following the response); a 200 ms window (from -200 to 0 ms prior to stimulus onset) served as the baseline.

For the face recognition task, the VPP component was scored as the maximum amplitude in the 150–200 ms time-window over Cz electrodes site; N170 was scored as the minimum amplitude in the 150–200 ms time-window over O1, O2, P7, and P8 electrodes sites. For the passive picture viewing, EPN was scored as the minimum amplitude in the 200–300 ms time-window and LPP was scored as the mean amplitude in the 400–1,000 ms time-window (both were measured at CPz electrodes site).

## Results

### Stimuli Ratings

The ratings of IAPS pictures and faces (**Table 2**) were submitted to independent repeated measures ANOVAs with group (two levels: burnout and control) and stimulus type (three levels: neutral, negative, positive) factors. There was no significant between-group difference for IAPS pictures [*F*_(1,91)_ = 0.266, *p* = 0.607] or faces [*F*_(1,91)_ = 2.406, *p* = 0.124]. No significant difference between the burnout group and the control group was observed.

**Table 2:** The means (M) and standard deviations (SD) for the ratings of IAPS pictures and faces for the burnout and control group (ratings refer to a scale ranging from 1-very negative to 9-very positive).

	Burnout (*N* = 47)	Control (*N* = 46)
	*M (SD)*	*M (SD)*
**IAPS**		
Neutral	5.19 (0.59)	5.23 (0.54)
Negative	2.21 (0.71)	2.05 (0.80)
Positive	7.29 (1.03)	7.54 (0.84)
**FACES**		
Neutral	4.94 (0.35)	6.75 (0.94)
Negative	3.74 (0.93)	3.67 (0.96)
Positive	6.29 (1.08)	5.01 (0.65)


There were significant differences in the ratings of the IAPS pictures [*F*_(2,182)_ = 957.97, *p* < 0.0001] and faces [*F*_(2,182)_ = 234.60, *p* = 0.0001] between all stimulus categories (neutral, negative, and positive). No interaction effects were found.

### Psychophysiological Results of Face Categorization Task

The values of N170 (separately extracted from O1, O2, P7, and P8 electrodes sites) and VPP (extracted from Cz electrodes site) were submitted to independent repeated measures ANOVAs with group (two levels: burnout and control) and stimulus type (three levels: neutral, distorted, and emotional faces) factors. In all the performed analyses, faces with positive and negative facial expressions were placed in one category called “emotional faces”; thus, instead of four stimulus types, we operated on three separate categories: neutral, emotional, and distorted.

#### N170 ERP Component

The analysis of N170 showed no between-group difference, [O1 electrodes site: *F*_(1,91)_ = 0.064, *p* = 0.801; O2 electrodes site: *F*_(1,91)_ = 0.025, *p* = 0.875; P7 electrodes site: *F*_(1,91)_ = 0.082, *p* = 0.776; P8 electrodes site: *F*_(1,91)_ = 0.166, *p* = 0.685], thus we did not explore the N170 component further.

#### VPP ERP Component

**FIGURE 2:**
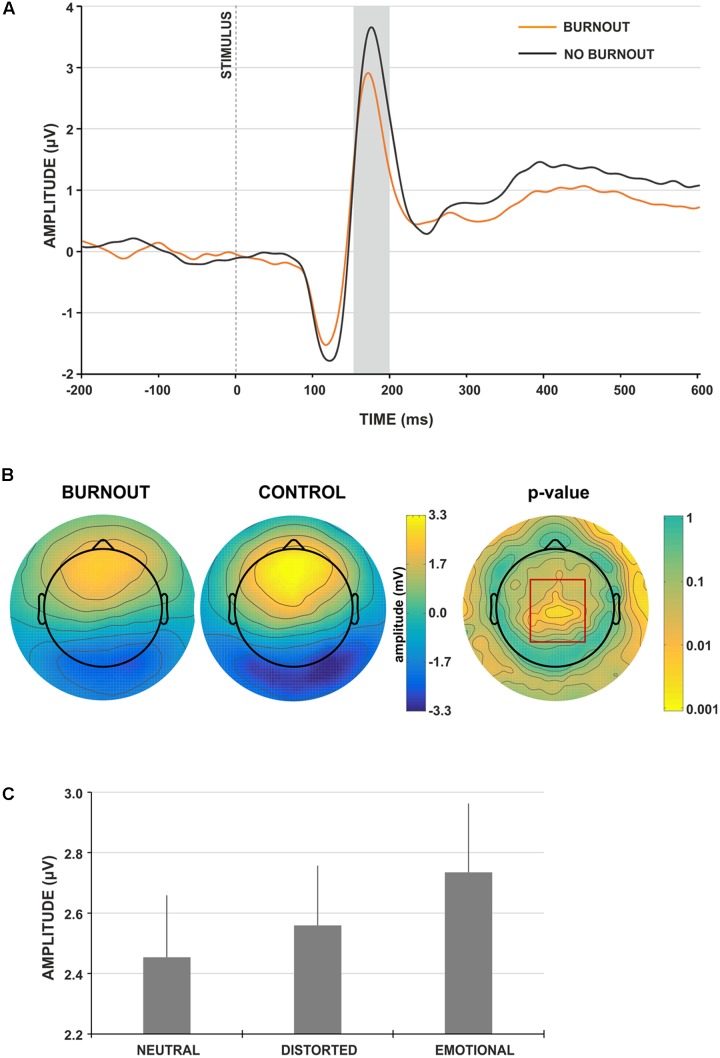
Face-locked ERP characteristics. **(A)** Grand-average face-locked VPP component at electrode site Cz for burnout (orange line) and control (gray line) group. The gray rectangle denotes the time-window selected for the ERP analyses. **(B)** Scalp topography for the time-window of the VPP component for burnout and control group. The red rectangle denotes the region showing significant between-group difference. **(C)** Grand-average amplitudes of the VPP component for neutral, distorted and emotional faces. Vertical bars denote standard errors.

The analysis of the VPP component revealed the main effect of group type; the amplitude of the VPP component was significantly lower for participants presenting burnout syndrome [*F*_(1,91)_ = 7.546, *p* = 0.007, $\eta_{p}^{2}$ = 0.077; **Figures 2A,B**). **Figure 2A** presents the grand-average face-locked VPP component at electrode site Cz, where significantly lower VPP amplitude in burnout subjects is observed. The lower VPP activity in burnout is also illustrated on topographic activity maps (**Figure 2B**).

Moreover, the main effect of stimulus type was observed [*F*_(2,182)_ = 6.016, *p* = 0.003, $\eta_{p}^{2}$ = 0.062]. The *post hoc* analysis showed that the amplitude of VPP for emotional faces was significantly higher (**Figure 2C**), compared to neutral and distorted faces (*p* < 0.001 and *p* < 0.05, respectively). No interaction effect of group and stimulus type was observed.

To explore further the relationship between the amplitude of the VPP component for different stimuli types, correlation analysis with MBI subscales was performed; this showed a consistent, statistically significant relation between VPP amplitude for all stimuli types and MBI-cynicism scores (**Table 3**). Namely, the higher the MBI-cynicism score, the lower the VPP amplitude for neutral, distorted and emotional faces.

**Table 3:** Pearson correlation coefficients (*r*) between subscales of MBI-GS and amplitude of VPP component in face categorization task in three categories of stimuli: neutral, distorted and emotional.

	Categories of stimuli		
	
	Neutral	Distorted	Emotional
**MBI-GS subscales**			
Exhaustion	-0.177	-0.222^∗^	-0.156
Cynicism	-0.291^∗^	-0.229^∗^	-0.243^∗^
Efficacy	0.174	0.109	0.168


*Asterisks denote statistical significance (^∗^*p* < 0.05).*

### Psychophysiological Results of the IAPS Task

The values of the EPN and LPP components extracted from CPz electrodes were submitted to independent repeated measures ANOVAs with group (two levels: burnout and control) and stimulus type (three levels: neutral, positive, and negative) factors.

**FIGURE 3:**
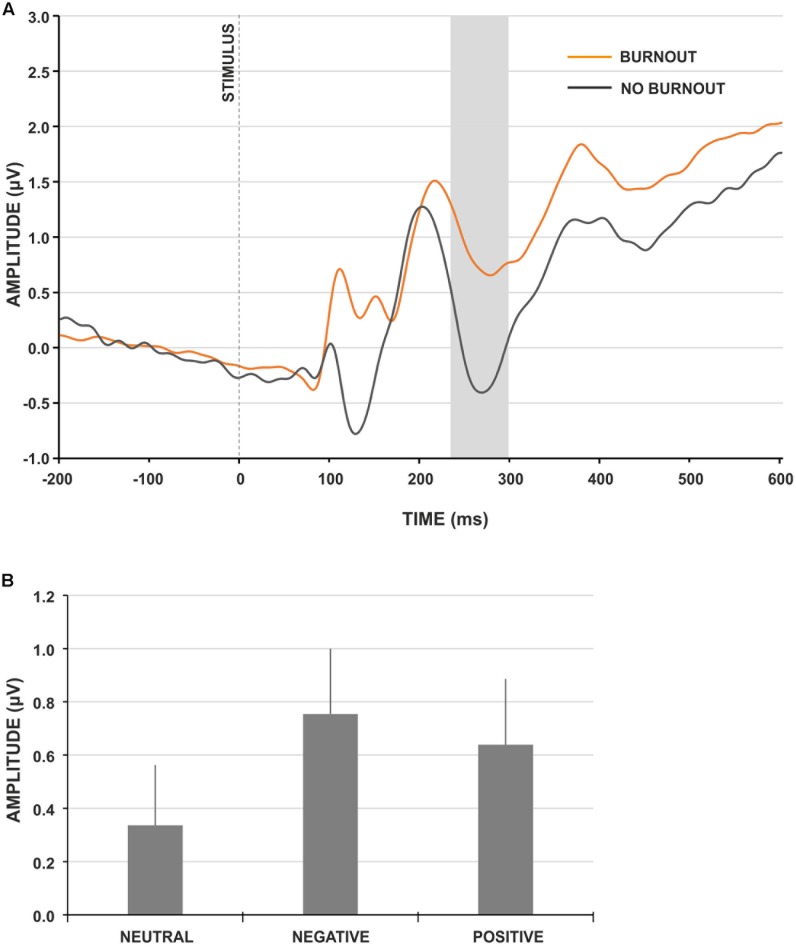
IAPS-locked ERP characteristics. **(A)** Grand-average IAPS-locked EPN component at electrode site CPz for burnout (orange line) and control (gray line) group. The gray rectangle denotes the time-window selected for the ERP analyses. **(B)** Grand-average amplitudes of the EPN component for neutral, negative and positive IAPS pictures. Vertical bars denote standard errors.

The LPP component was not sensitive to the group factor [*F*_(1,91)_ = 0.381, *p* = 0.538, $\eta_{p}^{2}$ = 0.004]; thus, it was not analyzed further. For the EPN component, we observed the main effect of group type (**Figure 3A**) and the amplitude of EPN was significantly more negative for participants not presenting burnout syndrome [*F*_(1,91)_ = 4.195, *p* = 0.043, $\eta_{p}^{2}$ = 0.044]. Moreover, the main effect of stimulus type was observed [*F*_(2,182)_ = 8.892, *p* < 0.001, $\eta_{p}^{2}$ = 0.089]. The *post hoc* analysis revealed that the amplitude of EPN for neutral IAPS pictures was significantly less negative, compared to EPN for negative (*p* < 0.001) and positive (*p* < 0.05) IAPS pictures (**Figure 3B**). No interaction effect of group and stimulus type was observed.

To explore further the relationship between the amplitude of the EPN component for different stimuli types and burnout syndrome, correlation analysis with MBI subscales was performed: it showed a consistent, statistically significant relation between the EPN amplitude for negative and positive IAPS pictures and MBI exhaustion and cynicism scores (**Table 4**). Namely, the higher the MBI exhaustion and cynicism scores, the less negative the EPN amplitude for negative and positive IAPS pictures.

**Table 4:** Pearson correlation coefficients (*r*) between subscales of MBI-GS and amplitude of EPN component for three categories of IAPS pictures: neutral, negative, and positive.

	Categories of IAPS pictures		
	Neutral	Negative	Positive
**MBI-GS subscales**			
Exhaustion	0.172	0.226^∗^	0.229^∗^
Cynicism	0.174	0.229^∗^	0.206^∗^
Efficacy	-0.092	-0.111	-0.125


*Asterisks denote statistical significance (^∗^*p* < 0.05).*

## Discussion

The study results partially confirm the presented hypotheses. Burnout subjects demonstrate a weaker response to affect-evoking stimuli (general hypothesis); this is indexed by a decline in VPP amplitude to emotional faces (hypothesis 1) and decreased EPN amplitude in processing emotional scenes (hypothesis 2). The assumption of a possible decrease in LPP amplitude in response to negative stimuli in burnout subjects (hypothesis 3) cannot be supported in the light of the presented results.

In face categorization tasks, the decrease of VPP amplitude indicated weaker neurophysiological response in burnout subjects, compared to healthy controls. A decreased response was observed in each face stimuli category (emotional, neutral, and distorted). This outcome is consistent with the study of Tei et al. (2014), in which they showed that burnout is associated with reduced activity in empathy-related brain areas, weakened emotional regulation and difficulty in recognizing emotional state. As VPP is related to the perceptual processing and structural encoding of facial stimuli, the decreased VPP may indicate a weaker response to face stimuli among burnout subjects. This might have further social consequences in work-related contexts and may be related to weaker responses to emotional contexts, which appear to be the core characteristic of cynicism and depersonalization. However, it is worth noting that when assessing pictures as negative, positive, or neutral, the rating scores of participants with burnout did not differ from those of the controls. Thus, careful and conscious perception of face expressions seems to be intact in burnout individuals.

This tendency is also observed in passive viewing of natural scenes in three stimuli categories: positive, negative, and neutral scenes. The response to natural scenes (indexed by EPN) was significantly weaker (less negative) in the burnout group. As EPN is related to automatic attentional allocation (Schupp et al., 2006), it may be hypothesized that in the studied tasks the costs of long-term work-related stress are observed in very early automatic and unconscious stages.

Thus, similar conclusions emerge for the two kinds of affect-related stimuli (faces and natural scenes) in the two types of affective information processing (active and passive): the initial, early phase of affective information processing is impaired and weakened. On the contrary, LPP (described as selective attention toward motivationally salient information, representation of stimuli in working memory (Donchin and Coles, 1988) and as a neural marker of emotion regulation (Qi et al., 2016) did not reveal any differences. Again, the electrophysiological results are in line with the data obtained from the rating of pictures. The similar rating results of IAPS pictures shows that there was no significant difference between the burnout group and the control group on the level of conscious perception. The obtained results lead to a similar observation as was found in error processing. The differences in the very early stages of information processing may not influence further processing, but may be related to the additional effort required to achieve the same results. This may suggest the compensatory effort that we described in our previous findings and may lead to similar conclusions about the possible “hidden costs” of burnout (Golonka et al., 2017).

Comparing the presented findings with our previous study on cognitive impairments in burnout (Golonka et al., 2017), an interesting aspect may be highlighted: in the case of external affect-related stimuli, the response in the early stage of information processing is weaker among burnout subjects, compared to healthy controls. This tendency changes if the subject refers to internal and individual contexts when committing an error: the early phase of information processing is significantly enhanced (indexed by increased error related negativity amplitude—ERN). In this situation, the decrease in neurophysiological response is observed later (indexed by the lower amplitude of the late positivity ERP component—Pe); this could possibly lead to difficulties in proactive control and active goal maintenance.

The decrease in neurophysiological response observed among burnout subjects when processing emotional information may be particularly interesting in the context of correlation analyses (see **Tables 3**, **4**). The correlations consistently show a significant association between analyzed ERP components and two core burnout symptoms (emotional exhaustion and cynicism), but not with efficiency. The amplitude of VPP is negatively related to cynicism in all stimuli categories (neutral, distorted and emotional) and with emotional exhaustion in distorted faces: the higher the burnout scores, the weaker the neurophysiological response. Similarly, EPN is associated with emotional exhaustion and cynicism: the correlation coefficients with negative and positive IAPS stimuli are positive, but—keeping in mind that we are analyzing negative depletion—it may be assumed that the higher the burnout scores on exhaustion and cynicism, the less negative the EPN component that is observed.

In the context of the important debate on the difficulties related to differentiating burnout from closely related states and disorders (mainly depression and anxiety, e.g., Korczak et al., 2010; Golonka et al., 2014; Bianchi and Laurent, 2015), it is particularly important to discuss discriminative points. Emotion processing in burnout and depression show some common features: in both cases, the response to affect-evoking stimuli is weaker. However, from a more detailed perspective, we can indicate some important differences: the deficits in emotion processing are noticed only in the early stage of this process (decreased VPP, EPN), but not in further processing (no changes in LPP), while in depression both components are amplified. Interestingly, in the case of the LPP component, burnout remains “in between” anxiety and depression: anxious individuals tend to demonstrate larger LPP amplitude, while individuals with clinical depression demonstrate the opposite (Hajcak, 2012).

Regarding the limitations of the study, the weaker neurophysiological response that is observed in the burnout group may be considered both as a burnout marker and as the result of changes observed on behavioral and subjective levels, i.e., specific attitudes and behaviors linked to emotional exhaustion, cynicism and depersonalization. This is still an essential issue in burnout research. An answer may emerge from longitudinal studies, but considering the complexity and fluctuating nature of burnout syndrome, it is highly problematic to design a longitudinal study on burnout.

## Conclusion

The obtained results are further evidence for impaired stimuli processing in individuals presenting burnout symptoms. Burnout subjects reveal impairments in early stages of emotion information processing in two different categories of stimuli: faces and natural scenes.

The correlation analyses revealed that ERP components related to emotional processing (VPP and EPN) are associated with two core burnout symptoms: emotional exhaustion and cynicism.

Event-related potentials that differentiate processing of affect-related information between burnout and controls (VPP and EPN) may be potential indicators of burnout syndrome. We hypothesize that the decreased amplitude of VPP and EPN components in the burnout group may be the neurophysiological manifestation of emotional blunting and may be considered as neurophysiological markers of emotional exhaustion and cynicism. Additionally, LPP amplitude, which does not differ between burnout individuals and controls, may be a diagnostic criterion that differentiates burnout from depression and anxiety disorders.

The presented findings contribute to depicting neurophysiological outcomes as indicators of burnout syndrome. This might help to improve the diagnostic process of burnout and lead to a more precise description of the cognitive and emotional mechanisms underlying burnout syndrome.

## Author Contributions

KG and MG: substantial contributions to the conception and design of the work; acquisition, analysis, interpretation of data, drafting the work and revising it critically; final approval of the version to be published; agrees to be accountable for all aspects of the work. JM-K: substantial contributions to the conception and design of the work; analysis, interpretation of data, drafting the work and revising it critically; final approval of the version to be published; agrees to be accountable for all aspects of the work. KP: substantial contributions to acquisition, analysis, drafting the work and revising it critically; final approval of the version to be published; agrees to be accountable for all aspects of the work. TM: substantial contributions to the conception and design of the work; drafting the work and revising it critically; final approval of the version to be published; agrees to be accountable for all aspects of the work.

## Conflict of Interest Statement

The authors declare that the research was conducted in the absence of any commercial or financial relationships that could be construed as a potential conflict of interest.

## Footnotes

1 The IAPS pictures used were **pleasant** (1050, 1200, 1300, 2730, 2800, 3010, 3160, 3170, 3230, 3261, 3300, 3350, 6200, 6210, 6230, 6244, 6250, 6312, 6313, 6370, 6550, 6560, 6571, 6821, 9040, 9042, 9050, 9253, 9300, 9400, 9405, 9410, 9433, 9520, 9600, 9611, 9810, 9910, 9920, 9921), **neutral** (2190, 2320, 2570, 2840, 2880, 5390, 5532, 5534, 5731, 5740, 5800, 5900, 7000, 7002, 7004, 7006, 7009, 7010, 7025, 7034, 7035, 7040, 7041, 7060, 7080, 7090, 7100, 7130, 7140, 7150, 7175, 7190, 7217, 7224, 7233, 7235, 7491, 7550, 7595, 7950), and **unpleasant** (1463, 1601, 1710, 1811, 2000, 2070, 2080, 2091, 2092, 2165, 2340, 2345, 4002, 4290, 4532, 4572, 4608, 4658, 4659, 4660, 4664, 4810, 5470, 5621, 5626, 5628, 7325, 8021, 8032, 8080, 8200, 8210, 8280, 8320, 8370, 8400, 8461, 8465, 8490, 8540) (Foti et al., 2009).

2 http://sccn.ucsd.edu/eeglab
